# Situational awareness and information flow in prehospital emergency medical care from the perspective of paramedic field supervisors: a scenario-based study

**DOI:** 10.1186/s13049-014-0083-x

**Published:** 2015-01-16

**Authors:** Teija Norri-Sederholm, Heikki Paakkonen, Jouni Kurola, Kaija Saranto

**Affiliations:** Department of Health and Social Management, University of Eastern Finland, PL 1627, Kuopio, 70211 Finland; Centre for Pre-hospital Emergency Care, Kuopio University Hospital, PO Box 1777, Kuopio, 70210 Finland

**Keywords:** Prehospital emergency care, Critical information, Paramedic field supervisor, Information flow, Situational awareness

## Abstract

**Background:**

In prehospital emergency medical services, one of the key factors in the successful delivery of appropriate care is the efficient management and supervision of the area’s emergency medical services units. Paramedic field supervisors have an important role in this task. One of the key factors in the daily work of paramedic field supervisors is ensuring that they have enough of the right type of information when co-operating with other authorities and making decisions. However, a gap in information sharing still exists especially due to information overload. The aim of this study was to find out what type of critical information paramedic field supervisors need during multi-authority missions in order to manage their emergency medical services area successfully. The study also investigated both the flow of information, and interactions with the paramedic field supervisors and the differences that occur depending on the incident type.

**Methods:**

Ten paramedic field supervisors from four Finnish rescue departments participated in the study in January–March 2012. The data were collected using semi-structured interviews based on three progressive real-life scenarios and a questionnaire. Data were analysed using deductive content analysis. Data management and analysis were performed using Atlas.ti 7 software.

**Results:**

Five critical information categories were formulated: Incident data, Mission status, Area status, Safety at work, and Tactics. Each category’s importance varied depending on the incident and on whether it was about information needed or information delivered by the paramedic field supervisors. The main communication equipment used to receive information was the authority radio network (TETRA). However, when delivering information, mobile phones and TETRA were of equal importance. Paramedic field supervisors needed more information relating to area status.

**Conclusions:**

Paramedic field supervisors communicate actively with EMS units and other authorities such as Emergency Medical Dispatch, police, and rescue services during the multi-authority incidents. This study provides knowledge about the critical information categories when receiving and sharing the information to obtain and maintain situational awareness. However, further research is needed to examine more the information flow in prehospital emergency care to enable a better understanding of required communication in situational awareness formation.

## Background

One of the key factors in the successful delivery of prehospital emergency medical care is the efficient management and supervision of emergency medical services (EMS) units. In Finland this is the task of the paramedic field supervisor (PFS). They need to ensure an adequate number of EMS units in their designated area and they have an important EMS leadership role in cases where several EMS units are needed and/or multi-authority incidents [1,2]. PFS are required to make a great number of decisions rapidly, and in most cases, under pressure. These decisions depend on their situational awareness (SA) [3].

SA is derived from information and its interpretation: without having enough the right type of information, there is no situational awareness [4-6]. The most important information categories at PFS’s work were events, means, action patterns and decisions [7]. In order to support the accurate formation of SA, critical information needs should be identified [8]. Furthermore, when making decisions in multiple casualty incidents, good information flow is required: information is needed from different sources to create a correct mental picture of what is going on. Decisions based on low-grade information can lead to poor patient outcomes and/or risks to rescuers [3].

An important part of PFS work is the co-operation and sharing of information with other authorities involved in the incident event. This co-operation also enables shared situational awareness (SSA) [9]. Seppänen et al. [8] have collated the major factors that hampered the Search and Rescue (SAR) organisation in achieving adequate SSA. These influential factors were information gaps, the lack of fluent communication, and the fact that there was no common operational picture in use. They also found out that the factors affecting information gaps were agencies focusing only on their own tasks, unclear information delivery processes, shortages of incident information, agencies passivity, and a lack of up-to-date information.

The aim of this study was to find out what type of critical information paramedic field supervisors need during multi-authority incidents in order to manage their emergency medical services area successfully. The study also investigated the flow of information, with whom the paramedic field supervisors co-operate and how, and the differences that occur depending on the incident type.

## Methods

### Ethics

All rescue departments gave their permission for the research. Before the interview, all participants were informed about the study and signed Informed Consent Form including the description of study, the purpose of its use, the confidentiality, and the rights of the participant. The University of Eastern Finland Committee on Research Ethics approved the study on 15 December 2011.

### Questionnaire design

Three progressive scenarios based on real-life experiences were used in the study. The scenarios were selected to represent different types of prehospital incidents and the paramedic field supervisors’ leadership role in these incidents. The scenarios were written by the first author based on the discussions with the two co-authors from the Centre of Prehospital Emergency Care. The scenarios were pre-tested by two prehospital emergency care professionals using the same interview method as in the study. After pre-testing, changes were made according to the feedback. After that, another two informal pilot interviews were conducted by the corresponding author. The interviews involved an Emergency Response Centre (ERC) instructor and a police field commander, both of whom requested minor changes. These were implemented, and enhanced the validity of the scenarios. The validity check included both the content and the correctness of the work protocol and actions during the scenario.

The first scenario was a road traffic accident with eight potential patients. The accident took place in winter, approximately 30 km from the city centre, at a time when the paramedic field supervisor was in the city centre leading a team in resuscitation. The second scenario was set on a Saturday night in early June, at the start of the school summer holidays. Many young adults in multiple locations of one neighbourhood were feeling unwell and eventually lost consciousness; it was later revealed that they were members of a group of eight young adults who had bought cheap alcohol containing poisonous methanol from an unknown person. The third scenario involved a shooting threat outside of a shopping centre, which ended with one person being wounded. The situation required the presence of an ambulance unit in a safe zone.

### Selection of the study population

Ten paramedic field supervisors from four Finnish rescue departments volunteered to participate in the study. The rescue departments represented both different geographic areas of Finland and different sized organisations in order to obtain sample diversity.

### Interview method

The data were collected from January to March 2012, using semi-structured interviews and a questionnaire.

Interviews were conducted by the first author in the rescue departments at day time. During the interview the paramedic field supervisors were off-duty. The interviewer simulated the different authorities during the interview and the interviews were audio-recorded. The mean duration of the interviews was approximately 70 minutes. The scenarios proceeded in a realistic manner. Based on the practice in their area, the paramedic field supervisors received the information they would routinely receive from ambulance units or other authorities, such as the ERC, the rescue department and the police. Some information provided was intended for the paramedic field supervisors and some was not. During the interview, the paramedic field supervisors asked for more information from other field authorities as they would normally do in their daily work. In turn, they were given the information that was available at that particular step of the scenario. As the scenario proceeded, they made decisions, such as whether to participate in the incident in the field or not, and they delivered information to other authorities. At the same time, they had to maintain their normal duties to ensure that there were enough free resources in their area for other possible incidents. Some scenarios caused a situation where there were insufficient or no ambulances in the area for the incident. The interviewees were asked to describe what type of information they were looking for and why, what information they delivered to other agencies, and what were they thinking during the incident.

All interviewees volunteered to answer a questionnaire [10] after the interview. The questionnaire included three open questions: 1) What information is important to receive in relation to your actions? 2) What information is important to deliver to the other agencies? and 3) What information would you have liked more of?

### Data collation and analysis

Interview data were transcribed verbatim. The only changes made were to dialect words, which were changed to standard language to avoid identifying the area where the interview was conducted. The names used to recognise the area were also changed.

The data were analysed using content analysis, a research technique which, through the use of categories, draws replicable and valid inferences from texts in the context of their use [11,12]. This study applied Choo’s [13] information management model in creating the themes for analysis.

**Figure 1:**
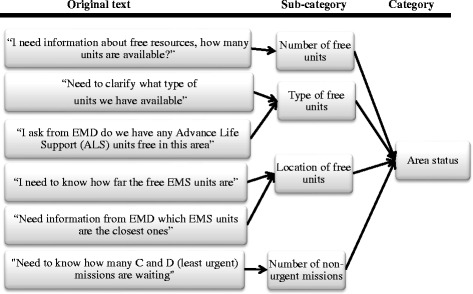
**An example of creating a category for information needs.**

The text was first coded into six themes created from the flow of information. The first three themes related to information needs: the kind of information paramedic field supervisors needed, from whom they received it, and through what communication device. The next three themes related to delivered information: the kind of information paramedic field supervisors delivered, to whom they sent it, and by what method. The coding was done using Atlas.ti 7 qualitative data software, and text belonging to the code could be either a meaningful complete sentence or a couple of words with a meaningful purpose. To increase reliability, the text was coded one scenario at a time. To ensure the validity of the coding, a check was done by the corresponding author after all the text was coded. The total number of codes was 684. The analysis continued by adding the data to an Excel spreadsheet to create the categories for each theme (Figure 1) based on the analysis. The findings were changed to percentages to enable comparison.

The total number of questionnaire findings was 129. The number of items relevant to receiving information was 62, 44 items were relevant to delivering information, and 23 data items related to the need for more information. The narrative text was content analysed and categorised in the same manner as the interview data to enable comparison.

## Results

Findings relating to information flow and critical information categories, and their differences between the scenarios, are first described. This is done category by category, relaying the findings for both information needed and information delivered at the same time. The findings of the questionnaire are then explained.

### Critical information categories

Five critical information categories were identified from the data: Incident data, Mission status, Area status, Safety at work, and Tactics. Incident data was the most important critical information category both in needed and delivered information (Table 1); this result was the same in all three scenarios. The second category varied depending on the scenario. In the traffic accident scenario, Mission status was the second most common category for both needed and delivered information, whereas in the youth scenario Area status was most needed and Tactics in delivered information. In the shooting scenario, Safety at work stood out, although Mission status was also important in delivering information for such incidents.

**Table 1:** **Information needed and delivered (%)**

**Category**	**Information needed**			**Information delivered**			**Needed**	**Delivered**
	**Scenario**			**Scenario**				
	**Accident**	**Youth**	**Shooting**	**Accident**	**Youth**	**Shooting**	**All**	**All**
	**(n = 113)**	**(n = 60)**	**(n = 52)**	**(n = 63)**	**(n = 27)**	**(n = 54)**	**(n = 225)**	**(n = 144)**
Incident data	44	51	42	67	70	39	46	57
Mission status	39	14	4	18	0	20	24	15
Area status	14	27	10	8	11	11	16	10
Safety at work	1	5	27	2	0	19	8	8
Tactics	2	3	17	5	19	11	6	10
TOTAL %	100	100	100	100	100	100	100	100

### Incident data

The critical information the paramedic field supervisors needed related to Incident data (Table 2) were preliminary knowledge (information based on an emergency call) about the incident, the number and status (triage) of patients, and detailed information received from agencies on the scene, for example, whether anyone was trapped in the road traffic accident case. They also needed specific information about the incident, such as information from the police about how many people were in danger or whether all the people involved were youths. In this category, almost half of the needed information related to detailed information. This information was mainly received via TETRA from EMS units on the scene. The typical Incident data information that paramedic field supervisors delivered to EMS units were orders, detailed information, and information received from other agencies, such as the police. They delivered the number of patients and detailed information about the incident to fire rescue and the police, and they also related the status of the patients to the police. The information delivered to the EMS doctor was mainly detailed information about the incident. Paramedic field supervisors made the preliminary notification to the hospital and gave detailed information about the incident.

**Table 2:** **Incident data details (%)**

**Incident data**			
**Information needed (n = 103)**	**%**	**Information delivered (n = 84)**	**%**
Detailed information from actors on scene (reason/cause, Anyone trapped?, estimated time for extrication)	48	Detailed information to EMS units, EMS doctor, rescue service and police	43
Status of patients	15	Action plan to EMS unit and to EMS doctor	21
Number of patients	14	Preliminary notification to hospital	13
Confirmation (mission code correct, medication given according to the protocol, are you ok?, police and rescue has the same number of patients, all patients checked, shooter is caught)	9	Number of patients to rescue and police	8
Preliminary knowledge from emergency call (what has happened, driving speed)	7	Detailed information to hospital, poison information centre, and telephone health service	8
Specific information about incident (How many people in danger? What kind of gun? Where are other possible patients? Are all involved persons young people?	7	Status of patients to police	5
		Confirmation to EMD	1
		Patient data to EMS report	1

### Mission status

Information needs relating to Mission status (Table 3) varied depending on the scenario. The typical information needs were both the number and skill level of EMS units assigned to the mission, and information on whether the assigned resources were sufficient. Paramedic field supervisors also needed other information, such as whether an EMS doctor was already assigned to the mission, when the EMS units could be released, and the estimated action time. They delivered information, such as their own status (on the way/estimated time of arrival at the scene, on scene, not available), the sufficiency of the EMS units (enough, need more, can be released), the estimated action time, and confirmation of a completed mission.

**Table 3:** **Mission status details (%)**

**Mission status**			
**Information needed (n = 53)**	**%**	**Information delivered (n = 21)**	**%**
EMS units assigned to the mission	41	Sufficiency of the EMS units	29
EMS resources	26	Estimated action time	14
Estimated action time	7	Mission completed	14
EMS doctor assigned to mission	7	PFS at scene	9
Location of the EMS units on the way to the mission	6	PFS Estimated arrival time to the scene	9
Hospital capability of admitting patients to be confirmed	4	PFS and EMS doctor not available to the mission	5
Number of police units assigned to mission	4	EMS doctor joined the mission	5
Distance to hospital	1	Decision of EMS unit to join another mission	5
Guidance to the scene	1	Request to EMD to add PFS to the mission	5
Can we change the units?	1	Change of the mission urgency	5
Possibility to free any EMS unit	1		
Mission completed	1		

### Area status

Typical information needs for Area status (Table 4) were the number, type, and location of free EMS units, the status and location of the occupied units, the availability of an EMS doctor, and the possibility of getting more units (created ad hoc or from the neighbouring town). The youth scenario had the highest Area status value. Paramedic field supervisors delivered information relating to Area status mainly to Emergency Medical Dispatch (EMD). It was about giving instructions on how to manage urgent and non-urgent incidents and their current status to identify the possibility to free some EMS units if needed, and the PFS availability.

**Table 4:** **Area status details (%)**

**Area status**			
**Information needed (n = 37)**	**%**	**Information delivered (n = 14)**	**%**
Available EMS units (number, type, location)	24	PFS available if needed	22
Availability of EMS doctor	22	Action plan to EMD how to manage urgent and non-urgent missions	22
Status of missions in the area	19	EMD to stop non-urgent missions for a moment	14
Location of occupied units	11	Info to rescue service that most probably first response missions will increase	7
Number of non-urgent missions	8	EMD to take care of area status	7
Possibility to have EMS units from neighbour town	8	EMD to inform EMS doctor about the shooting case	7
Possibility to create ad hoc EMS units	5	EMD to temporarily re-locate the EMS units to ensure the coverage in the area	7
Are missions connected?	3	EMD can deactivate ad hoc EMS units	7
		PFS not available	7

### Safety at work

In the Safety at work category (Table 5), paramedic field supervisors had a generic information need in all scenarios, this was a request to the police or rescue services on whether there were any safety risk factors. The rest of the needs related to the shooting scenario; which were the location of the safe zone and permission to enter the scene. Universally the paramedic field supervisors only accepted this information from the police in charge of the operation. After receiving the required information in the shooting scenario, the PFS cascaded the safety action plan to all EMS units and the EMS doctor.

**Table 5:** **Safety at work details (%)**

**Safety at work**			
**Information needed (n = 18)**	**%**	**Information delivered (n = 11)**	**%**
Location of safe zone	61	Safety plan to EMS units and EMS doctor	55
Any risk factors?	28	Information about safety risk to all EMS units in the area	18
Permission to go to the scene	11	Information about no safety risk anymore to all EMS units	18
		Information about safety risk to rescue service	9

Tactical information (Table 6) needs in the shooting scenario were received mainly from the police. In the road traffic accident and youth scenarios, the information needs related to operative leadership. This information came from the EMS doctor and the EMS unit currently in charge of the incident. After analysing the information received, paramedic field supervisors made the tactical action plan and passed it on to the units, doctor and the police.

**Table 6:** **Tactics details (%)**

**Tactics**			
**Information needed** ** (n = 14)**	%	**Information delivered** **(n = 14)**	%
Instructions how to act from police	64	Action plan to EMS units and EMS doctor	71
Opinion from police	22	Situation picture to police	22
Leadership relations	14	Action plan to police	7

### Information sources and targets

There were differences relating to information sources and targets, i.e., the social network, of the EMS (Table 7). Paramedic field supervisors mainly received information from the EMD and, depending on the case, from the police, EMS unit, and fire rescue. When looking at all the data, it seems that paramedic field supervisors both receive and deliver information to the EMS in equal measures. Paramedic field supervisors receive more information from the EMD and the police than they deliver back to those groups; however they mainly disseminate information to the EMS doctor, hospital, and fire rescue teams than receive information back.

**Table 7:** **Information sources and targets (%)**

**Source/Target**	**Received from**			**Delivered to**			**Received**	**Delivered**
	**Scenario**			**Scenario**				
	**Accident**	**Youth**	**Shooting**	**Accident**	**Youth**	**Shooting**	**All**	**All**
	**(n = 64)**	**(n = 34)**	**(n = 30)**	**(n = 41)**	**(n = 22)**	**(n = 41)**	**(n = 128)**	**(n = 104)**
EMS unit	24	30	7	17	32	27	23	24
EMS doctor	5	0	0	12	27	27	2	21
EMD	54	41	10	17	9	25	41	18
Fire rescue	11	0	0	22	4	7	6	13
Police	2	26	83	10	14	2	27	8
Hospital	2	0	0	22	14	12	1	16
Participants	2	3	0	0	0	0	2	0
TOTAL %	100	100	100	100	100	100	100	100

### Methods to receive and deliver information

As shown in Table 8, the paramedic field supervisors used three different methods to receive and deliver information. The use of communication equipment (TETRA, mobile phone) was the most common. However, there were differences in their use. Information was mainly received using TETRA, but when delivering information, TETRA and the mobile phone were used equally. Paramedic field supervisors used two information systems: the field command system and the electronic patient record. In this study, the field command system was only used to receive information. It is noteworthy that one quarter of the information was delivered face to face.

**Table 8:** **Methods used to receive and deliver information (%)**

**Method**	**Received**	**Delivered**
	**(n = 49)**	**(n = 34)**
Communication equipment	69	70
Authority radio network*	(63)	(35)
Mobile phone	(6)	(35)
Information system	22	6
Field command system	(16)	(0)
EPR	(6)	(6)
Face to face	9	24
TOTAL (%)	100	100

*Know by the acronym TETRA.

### Questionnaire results

In the questionnaire, Incident data was the most important information to receive and share; Mission status and Area status also featured prominently. Incident status, Safety at work, and Tactics were of similar importance when sharing the data. The paramedic field supervisors clearly needed more information relating to Area status. The Other category included information such as how the staff was coping, the channels in use, and the patient’s diagnosis in the hospital. In addition, there was also the comment that “I receive too much information”.

## Discussion

Five critical information categories were formulated: Incident data, Mission status, Area status, Safety at work, and Tactics. In general, the results indicate that paramedic field supervisors communicate actively. They mainly receive the information from other authorities via authority radio network (TETRA), but when it comes to delivering it, the use of TETRA and mobile phone was equally common.

The study provides knowledge about information sharing focusing on the information itself in prehospital emergency care. This aspect combined with analysis of multi-authority network and communication devices offers quite a unique set of research results in this domain. It increases the understanding of information flow, which can be taken into account in paramedic field supervisors’ daily work and education. The results also help in focusing to essential information needs in order to obtain and maintain situational awareness.

The five critical information categories identified in this study describe the paramedic field supervisors’ work quite well. Incident data, Mission status, Area status, Safety at work, and Tactics formulate the basic information for their daily activities. Their main task is to decide how the EMS units in the area are used and to support emergency medical dispatch (EMD) in cases when demand exceeds the supply of resources [1]. A literature review revealed very little on the question of the information aspect of PFS work. Since there is no prior research from this aspect, these results can be considered pioneering, which obviously makes referencing earlier findings very challenging. Safety at work and Tactics are usually mentioned in EMS textbooks, and they may even feature in a distinct chapter. However, the textbooks do not usually describe how to manage them in the entire EMS area.

The utmost importance of incident data in the paramedic field supervisors’ work was made apparent in this study. Almost half of the information needs and more than half of the delivered information related to incident data in all scenarios. It is also notable that almost half of the information needs related to detailed information. Paramedic field supervisors needed to know whether there were any special circumstances in the case that they should be aware of. However, they were not so interested in the details of patients’ clinical condition: the number of patients and their triage categories was sufficient, and the results from the questionnaire were in line with this. When delivering incident data, the paramedic field supervisors were clearly communicators between the EMS units and other authorities, sharing the information and thus enabling shared situational awareness (SSA).

Safety at work and Tactics in the road traffic accident were minor information needs, which was an unexpected finding. A possible explanation for this might be that in most of the cases, paramedic field supervisors did not take the lead in the situation. Not all of them went to the scene, and if they did, the fire rescue was chiefly responsible for the situation and an EMS unit was in charge of care; paramedic field supervisors did not want to interfere in the work itself. In the youth scenario, the safety information needs related to the scene-safety, such as the risk of the possible violence and the need to know if the police were already on-scene. The paramedic field supervisors did not deliver any safety related information. In the scenario, when it was found that the original reason for the cause of the unconsciousness and high respiration rate was an unknown liquid, presumably poisonous, the result was unexpected. In this kind of situation, it could be thought that PFS delivers or reminds EMS units about safety instructions. However, in the shooting scenario, Safety at work and Tactics had reasonably high information needs. This can be explained by the nature of the scenario.

The crucial role of communication within and among the teams and organisations to ensure safe clinical practice and effective organisational performance has only recently been recognised [14]. In general, it is important to understand the conformity and differences in and between the critical information categories to enable effective and reasonable communication during the incident. The results indicate that paramedic field supervisors communicate actively, although many of them felt overloaded with information. When aware of the critical information needs, it is possible to support the formation of situational awareness and focus on sharing the information elements needed to perform the core task [8]. This study highlights the key authorities with whom the PFS communicates and understanding this social network where paramedic field supervisors work in prehospital emergency care is a prerequisite for effective communication [15]. As mentioned earlier, information is needed from different sources in multiple casualty incidents to create an accurate mental picture of what is going on [3]. Effective information exchange is critical for developing good strategies as well as for accurate situational assessment. It also contributes to successful team performance [16]. The paramedic field supervisors’ role means that the information they deliver is essential in building SSA.

Interestingly, information received from other authorities was mainly received via authority radio network (TETRA). In delivering information, however, the use of TETRA and the mobile phone was equally common. The paramedic field supervisors generally used a mobile phone when calling the EMS doctor or police incident commander. It is also notable that the EMS units and EMS doctor received almost the same amount of information from the PFS. This raises a question that how much of this information was duplicated and is there a possibility to reduce the volume of communication if TETRA phone with common talk group is used. However, in this study there was no detailed comparison of that data. Since EMS involves teamwork, and because effective communication is the basis for excellence in emergency care, attention to communication devices is required [17]. Although not studied, mobile technology based on commercial networks might be vulnerable. This risk is partly covered by using TETRA network in communications between authorities. Information management systems were not a notable communication tool in this study because they were not in use in all of the areas participating in the study. However, further research is needed to find out what type of critical information is communicated (and could be communicated) on information management systems and if it could reduce the amount of radio traffic.

Further research is needed from the perspective of information to ensure both SA in PFS work and SSA in multi-authority incidents.

These findings can be used when creating field command information systems for prehospital emergency care, and when planning how to aggregate and view the information. Furthermore, the findings can also be used to develop PFS training.

### Limitations

Three limitations of the study have been identified. The findings are based on a specific Finnish EMS operation model [18] and this might be considered a limitation to the wider generalisation of the study. However, the same issues exist irrespective of the EMS system used. These findings can therefore be applied; although there may not be a similar system to organise EMS or use paramedic field supervisors, the same information needs and the need to share the information are universal. Another possible limitation of this study is the small sample size, since the study involved only ten paramedic field supervisors. However, they represented different sizes of organisations and different parts of Finland, and they had substantial experience in their roles. A third possible limitation is the fact that the data were not collected in real-life situations, possibly affecting the participants true responses. Nevertheless, the scenarios used in this study were created from real-life situations by a multidisciplinary team. The scenarios were also tested before the interviews took place.

## Conclusions

Paramedic field supervisors communicate actively with EMS units and other authorities like Emergency Medical Dispatch, police, and rescue services during the multi-authority incidents. This study provides knowledge about the critical information categories in receiving and sharing the information to obtain and maintain situational awareness. However, further research is needed to examine more the information flow in prehospital emergency care to enable a better understanding of needed communication in situational awareness formation.

## Abbreviations

EMD: Emergency Medical Dispatch
EMS: Emergency medical services
ERC: Emergency response centre
PFS: Paramedic field supervisor
SA: Situational awareness
SAR: Search and Rescue
SSA: Shared situational awareness
TETRA: Authority radio network
